# Treatment-seeking behaviour, awareness and preventive practice toward malaria in Abu Ushar, Gezira state, Sudan: a household survey experience from a rural area

**DOI:** 10.1186/s12936-022-04207-5

**Published:** 2022-06-11

**Authors:** Elfatih A. Hasabo, Rawan I. Khalid, Ghassan E. Mustafa, Ruaa E. Taha, Riham S. Abdalla, Reham A. Mohammed, Mazin S. Haroun, Rawaa Adil, Riham A. Khalil, Rawaa M. Mansour, Reham K. Mohamed, Heitham Awadalla

**Affiliations:** 1 Faculty of Medicine, University of Khartoum, Khartoum, Sudan; 2 Department of Community Medicine, Faculty of Medicine, University of Khartoum, Khartoum, Sudan

**Keywords:** Malaria, Awareness, Practices, Treatment-seeking behaviour, Sudan, Rural area

## Abstract

**Background:**

Usage of mosquito bed nets and the practice of other prevention methods are essential for the prevention of malaria in endemic areas. Proper community knowledge about malaria and prompt treatment-seeking behaviour for early diagnosis and treatment are crucial for eliminating the disease. This study aimed to assess the awareness, treatment-seeking behaviour, and prevention practices towards malaria in Abu Ushar, Gezira State, Sudan.

**Methods:**

A community-based, cross-sectional study was conducted in March 2021, including 310 households in Abu Ushar, Aljazeera, Sudan. Data were collected through face-to-face interviews with head of the household using an interviewer-administered questionnaire. Data were entered and analysed using R software.

**Results:**

A total of 310 households were enrolled in this study. Sixty per cent had children under the age of 5 years. The majority of these households (94.8%) had a history of malaria in the past 12 months. Overall, awareness of malaria was good; 197 (63.5%) households had bed nets in their houses; 75.8% of total households identified fever with shivering as a symptom of malaria. Regarding treatment-seeking behaviour, 77.9% seek treatment from the nearby primary health centre, and 60% seek treatment within the first day. Only 45.3% stated that everyone in the household sleeps under bed nets.

**Conclusion:**

High awareness about malaria and preventive measures was found among participants in households. Most households had previous infections with malaria. Therefore, an interventional programme should be established in this area to reduce this high rate of malaria.

## Background

Malaria is a public health problem and a common cause of death in Sudan [1]. Globally, It causes more than 400,000 deaths every year [2]. Most deaths occurred among young children and other high-risk groups, including pregnant women, non-immune travellers, refugees, displaced persons, and labourers in endemic areas [3].

Malaria is a major prevalent vector-borne disease within the world. It is threatening about 2–3 billion people in more than 90 countries, constituting 56% of the total population in the world [3]. In 2016, the World Health Organization (WHO) estimated 216 million malaria cases and 445,000 deaths due to malaria, and most of those deaths occurred within the African region [3]. Malaria caused by *Plasmodium falciparum* nearly accounted for 99% of African cases and 94% of all malaria cases and deaths globally in 2019 [3]. In Sudan, there were 2.4 million malaria cases in 2019, and it is considered the leading contributing country to malaria infection in the Eastern Mediterranean region, according to the WHO [3].

Furthermore, the development of drug resistance to the malarial parasite is a major threat to malaria control programmes in Sudan. Some surveys have reported the failure of artemisinin-based combination therapy (ACT) to treat uncomplicated malaria in Sudan, and a high prevalence of mutations in *P. falciparum* drug resistance genes was detected [3]. The study area in Abu Ushar, Gezira state carries a significant burden of malaria and partial indoor residual spraying to all households. Also, severe malaria cases represent 20% of the overall severe malaria caseload in the country and is the first cause of hospital admission. In 2016, 17% of total deaths were due to malaria in Gezira state [4], where malaria transmission is mainly seasonal with a fluctuating pattern. Almost all populations with low immunity are vulnerable to severe malaria, especially children under 5 years and pregnant women [5].

Health-seeking behaviour for malaria treatment is fundamental in reducing mortality. People are making some decisions after the onset of putative malaria symptoms, such as staying at home and doing nothing, treatment with herbal medication, and self-medication with drugs are considered bad treatment-seeking behaviour. Reports showed that some people still use self-medication, herbal therapy, and other informal treatment to manage malaria [6]: 41.2 and 23.9% in rural and urban areas, respectively, practiced self-treatment in Sudan. A study conducted in 2009 which covered rural areas of Elmatama and Malakal in Sudan reported limited practice of regular indoor residual spraying, ranging between 6.6 and 40% [7]. Among the communities being surveyed, a 3.7% usage of mosquito nets in Elmatama, and 49.6% in Malakal was reported; the proportions reported using mosquito nets the previous night were 63.0, 79.5, 35.8, and 42.6% in ElRank, Malakal, Elhosh, and ElMatama, respectively [7].

Although various attempts have been made to develop effective malaria vaccines, no immunization is yet commercially available, therefore prevention of malaria is currently by preventing mosquito bites. Other preventative measures include: the reduction of the parasite reservoir in the population in malaria-endemic regions; efforts to eradicate vectors (removal of breeding grounds, use of larvicides, insecticides; and, measures to reduce contact with the vector [8]. Countries remain free from malaria by being adherent to early diagnosis, appropriate treatment of the disease, integrated mosquito control programmes, and regular monitoring for drug resistance in *Plasmodium* species [2]. This study aimed to assess treatment-seeking behaviour, awareness and preventive practices among participants living in rural areas in Gezira state, Sudan. It is a malaria-endemic area, and there is a lack of solid evidence-based data regarding this topic.

## Methods

### Study area and participants

This study was conducted on 9 and 10 March, 2021 in Abu Ushar, Gezira state, Sudan during the rural residency programme for fifth-year medical students of the Faculty of Medicine, University of Khartoum. Abu Ushar is one of the rural areas in Gezira state, Sudan with surface area of 375 sq km, with a population of 122,354. It consists of 32 villages and 12 neighbourhoods. Each neighbourhood contains approximately 1900 persons. There are two hospitals and eight primary healthcare centres in Abu Ushar, and frequent malaria infections. Different preventive measures are used against malaria, such as cleaning houses, bed nets, window nets, and insecticides.

This study was conducted at household level in six of the 12 neighbourhoods. Not all neighbourhoods were covered because the study was conducted during the rural residency programme for the fifth-year medical students and a short time for data collection (2 days). Face-to-face interviews took place with heads of households. If the head of a household was not available, any responsible adult over 18 years was interviewed. All empty households or households without a responsible head were excluded.

### Study design and tool

A cross-sectional community-based study was conducted in Abu Ushar, Gezira state, Sudan at the household level. A structured quantitative questionnaire was used to assess household data, knowledge about malaria, treatment-seeking behaviour, usage of bed nets, and preventive practice towards malaria at household level. This pre-tested questionnaire was adapted from questionnaires of studies [9–12] and validated in previous studies among participants [9–12]. The questionnaire was prepared in English and then translated into Arabic, and a back translation was done for this questionnaire. Data collectors interviewed heads of the household in the local Arabic language. Piloting for this tool was done before data collection.

### Data collection and sampling

Eighteen data collectors were distributed in six neighbourhoods to interview and gather information from the head of household after introducing themselves, asking permission and explaining the objectives and importance of the study. Informed verbal consent was obtained from participants. The duration for collecting information was two days. Data collectors started interviews from early morning until evening, with short rest gaps. Six neighbourhoods were selected from 12 using an online random number generator, then a cluster sampling was used to reach households in each selected neighbourhood.

### Data quality

To ensure the quality of collected data, all data collectors were trained in communication skills to create a warm connection with families and households in the selected area to encourage heads of households to give accurate information related to the study. A pilot study to simulate the situation was conducted during collecting the data in households to find any problems in the questionnaire when interviewing began.

### Sample size

The sample size needed for this study was estimated using this formula:

$${\text{n = }}\frac{{{\text{z}}^{{2}} {\text{P(1}}{ - }{\text{P)}}}}{{{\text{d}}^{{2}} }}{.}$$

with a confidence interval (CI) = 95%, response distribution = 50% and margin of error = 0.05. The minimum required sample size was 384 households, but due to the short time available and the low number of data collectors, data collection was from 310 households.

### Data analysis

Quantitative data collected were coded and cleaned using Microsoft Excel and then analysed using R software version 4.0.2, presenting categorical data as percentages and counts. Continuous data were presented in tables as Mean ± standard deviation (SD) and Median (Interquartile range) (IQR) according to the skewness of data. Data were categorized according to ownership of bed nets within the household. Chi-square test and Fisher exact tests were used for categorical variables. An independent t-test was used for continuous variables to find any differences between the two groups according to the presence of bed nets.

## Results

### Household characteristics

A total of 310 households participated in this study, with a total of 1,955 members in all households. The mean of household members was 6.3 ± 2.6. The majority of the households (60%) had children under the age of 5 years. Half (50.3%) had more than one family living in the same house (Table 1).

**Table 1:** Characteristics of the households and prevalence of malaria in the previous 12 months according to the presence of bed nets

Variables	Overall, N = 310^1^	Presence of bed nets within the household		p-value^2^
		Yes, N = 197^1^	No, N = 113^1^	
Household member size?				
Total number	1955	1265	690	
Mean ± SD	6.3 ± 2.6	6.4 ± 2.6	6.1 ± 2.5	0.3
Number of children under 5 years in the house?				
0	124 (40.0%)	77 (39.1%)	47 (41.6%)	> 0.9
1	93 (30.0%)	60 (30.5%)	33 (29.2%)	
2	64 (20.6%)	42 (21.3%)	22 (19.5%)	
3	23 (7.4%)	15 (7.6%)	8 (7.1%)	
4	3 (1.0%)	2 (1.0%)	1 (0.9%)	
5	1 (0.3%)	0 (0.0%)	1 (0.9%)	
6	2 (0.6%)	1 (0.5%)	1 (0.9%)	
Do you have more than one family in your house? (Yes)	156 (50.3%)	105 (53.3%)	51 (45.1%)	0.2
Prevalence of malaria within households in the previous 12 months:				
Had malaria infection	294 (94.8%)	186 (94.4%)	108 (95.6%)	0.7
No malaria infection	16 (5.2%)	11 (5.6%)	5 (4.4%)	
How many person infected with malaria in the previous 12 months				
Mean ± SD	4.0 ± 2.8	4.0 ± 2.9	4.0 ± 2.6	0.91
Proportion of member infected with malaria in the previous 12 months/total number of member within houushold				
Median (IQR)	67% (36%–100%)	60% (33%–100%)	71% (43%–100%)	0.11

^1^Data were presented as n (%); Mean ± SD, Median (IQR)

^2^Fisher's exact test; Pearson’s Chi-squared test; Two Sample t-test, Mann whitney U test

### Prevalence of malaria

The vast majority of study participants (94.8%) stated that they had a history of malaria infection in their households in the past 12 months. The mean of members infected with malaria was 4.0 ± 2.8 (CI: 3.688–4.312). The mean proportion of family members infected with malaria in the previous 12 months was 64%. No significant difference was found between groups with and without bed nets within the household (Table 1).

### Knowledge and awareness about malaria

All study participants (100%) stated that they had heard about malaria infection, and 86.8% were able to identify mosquitoes as the mode of transmission for malaria; 75.8% were able to identify fever with shivering as a symptom of malaria; 20% of them identified *Plasmodium* organisms as the causative agent (Table 2). When asked about the preventive measures for malaria, most participants (60%) mentioned insecticide-treated bed nets, and insecticide-based repellent sprays (42.4%). More than half (63.2%) said that drainage of stagnant water is a way to prevent mosquito breeding. Further information is shown in Table 2.

**Table 2:** Knowledge and awareness about malaria in household heads

Variables	N	Overall, N = 310^1^	Presence of bed nets within the household		p-value^2^
			Yes, N = 197^1^	No, N = 113^1^	
Knowledge and awareness about malaria:					
Heard of malaria (Yes)	310	310 (100.0%)	197 (100.0%)	113 (100.0%)	
Source of information about malaria:	310				
Home/neighbours		159 (51.3%)	99 (50.3%)	60 (53.1%)	0.6
Radio/TV/newspapers		52 (16.8%)	35 (17.8%)	17 (15.0%)	0.5
Hospital/pharmacy		32 (10.3%)	21 (10.7%)	11 (9.7%)	0.8
Health workers		54 (17.4%)	35 (17.8%)	19 (16.8%)	0.8
I suffered from malaria		147 (47.4%)	94 (47.7%)	53 (46.9%)	0.9
Mode of transmission for malaria?	310				0.3
I do not know		27 (8.7%)	13 (11.5%)	14 (7.1%)	
Mosquito		269 (86.8%)	93 (82.3%)	176 (89.3%)	
Mosquito anopheles		7 (2.3%)	3 (2.7%)	4 (2.0%)	
Others		7 (2.3%)	4 (3.5%)	3 (1.5%)	
Causes of malaria:	310				0.3
Bacteria		15 (4.8%)	10 (5.1%)	5 (4.4%)	
Dirt/stagnant water		32 (10.3%)	25 (12.7%)	7 (6.2%)	
Germs		42 (13.5%)	30 (15.2%)	12 (10.6%)	
I do not know		25 (8.1%)	14 (7.1%)	11 (9.7%)	
Others		93 (30.0%)	54 (27.4%)	39 (34.5%)	
*Plasmodium* organisms		62 (20.0%)	36 (18.3%)	26 (23.0%)	
Virus		41 (13.2%)	28 (14.2%)	13 (11.5%)	
Symptoms of malaria:	310				
Headache		286 (92.6%)	182 (92.9%)	104 (92.0%)	0.8
Fever with shivering		235 (75.8%)	147 (74.6%)	88 (77.9%)	0.5
Fever with intervals		73 (23.5%)	50 (25.4%)	23 (20.4%)	0.3
Remission of fever with sweat		26 (8.4%)	17 (8.6%)	9 (8.0%)	0.8
Vomiting		163 (52.6%)	105 (53.3%)	58 (51.3%)	0.7
Weakness		206 (66.5%)	130 (66.0%)	76 (67.3%)	0.8
Loss of appetite		117 (37.7%)	72 (36.5%)	45 (39.8%)	0.6
Do not know		7 (2.3%)	3 (1.5%)	4 (3.5%)	0.3
Backache		109 (35.2%)	60 (30.5%)	49 (43.4%)	**0.022**
Others		175 (56.5%)	120 (60.9%)	55 (48.7%)	**0.036**
Knowledge about preventive measures of malaria:	309				
Untreated bed nets		176 (57.0%)	117 (59.7%)	59 (52.2%)	0.2
Insecticide treated bed nets		186 (60.2%)	125 (63.8%)	61 (54.0%)	0.09
Window nets		42 (13.6%)	26 (13.3%)	16 (14.2%)	0.8
Using insecticides based repellents sprays		131 (42.4%)	90 (45.9%)	41 (36.3%)	0.1
Cleaning of positive mosquitoes breeding and resting places		91 (29.4%)	68 (34.7%)	23 (20.4%)	**0.008**
Using mosquito coil/repellents		108 (35.0%)	72 (36.7%)	36 (31.9%)	0.4
Treatment (prophylaxis)		25 (8.1%)	14 (7.1%)	11 (9.7%)	0.4
Do not know		36 (11.7%)	16 (8.2%)	20 (17.7%)	**0.012**
Knowledge about ways to prevent mosquito breeding:	310				
Cleaning of house surrounding		185 (59.7%)	120 (60.9%)	65 (57.5%)	0.6
Draining of stagnant water		196 (63.2%)	136 (69.0%)	60 (53.1%)	**0.005**
Putting oil on stagnant water		59 (19.0%)	40 (20.3%)	19 (16.8%)	0.5
Do not know		36 (11.6%)	14 (7.1%)	22 (19.5%)	**0.001**
Others		45 (14.5%)	30 (15.2%)	15 (13.3%)	0.6

^1^Data were presented as n (%)

^2^Fisher's exact test; Pearson's Chi-squared test

Bold numbers in the p-value column mean they are statistically significant

Households with bed nets showed a significant difference in the following variables: knowledge of preventive measures, such as cleaning mosquito breeding and resting sites (P = 0.008) and draining stagnant water (P = 0.005) when compared with households without bed nets. However, households without bed nets in their house were more knowledgeable about backache as a symptom of malaria (P = 0.022). Households not having bed nets did not know about the preventive measures for malaria (P = 0.012) and ways to prevent mosquito breeding (P = 0.001) when compared with households with bed nets (Table 2).

### Treatment-seeking behaviour

Most participants (77.9%) stated that the primary health centre is where they seek treatment when they get symptoms of malaria. Most (69.1%) said that they seek treatment on the same day of presenting symptoms (Table 3).

**Table 3:** Treatment-seeking behaviour for malaria in households

Variables	N	Overall, N = 310^1^	Presence of bed nets within the household		p-value^2^
			Yes, N = 197^1^	No, N = 113^1^	
Treatment-seeking behaviour					
Has anyone in your household been ill with fever or malaria in the last 12 months? (Yes)	310	291 (93.9%)	189 (95.9%)	102 (90.3%)	**0.045**
If you or anyone in the household presented with signs and symptoms of malaria where would you seek treatment?	308				
Hospital		74 (24.0%)	50 (25.5%)	24 (21.4%)	0.4
Primary health centre		240 (77.9%)	153 (78.1%)	87 (77.7%)	> 0.9
Private clinic		7 (2.3%)	4 (2.0%)	3 (2.7%)	0.7
Pharmacy		46 (14.9%)	27 (13.8%)	19 (17.0%)	0.5
Drugs or local medication at home		13 (4.2%)	10 (5.1%)	3 (2.7%)	0.4
Herbal medicine		29 (9.4%)	16 (8.2%)	13 (11.6%)	0.3
Laboratory		10 (3.2%)	8 (4.1%)	2 (1.8%)	0.3
Did not seek treatments		6 (1.9%)	3 (1.5%)	3 (2.7%)	0.7
After how many days will you start seeking for treatments?	307				0.3
On the same day		212 (69.1%)	131 (67.2%)	81 (72.3%)	
After 1–3 days		90 (29.3%)	61(19.9%)	29 (9.4%)	
After 4–5 days		2 (0.6%)	1 (0.3%)	1 (0.3%)	
After 9–10 days		3 (0.9%)	2 (0.6%)	1 (0.3%)	

^1^Data were presented as n (%)

^2^Fisher’s exact test; Pearson’s Chi-squared test

### Usage of bed nets

More than half of the participants (63.5%) mentioned that they have bed nets available for use in their household, with a mean number of 3.0 ± 2.2 bed nets in each household; 72.6% stated that they obtained their bed nets from the market, while 31.5% got bed nets from a governmental institution (Table 4). Fewer than half of the participants (45.3%) stated that everyone in their household slept under the bed net in the past year. The main reason for not sleeping under bed nets was difficulty breathing while using the bed nets (18.1%) (Table 4).

**Table 4:** Usage of bed nets and reasons for not sleeping under bed nets in households

Variables	N	N = 310^1^
Usage of bed nets and practices of preventive measures:		
Does your household have any mosquito nets that can be used while sleeping? (Yes)	310	197 (63.5%)
How many ed nets your households have?	197	
Mean ± SD		3.0 ± 2.2
Proabortion of number of mosquito nets to the total number of household	197	
Mean ± SD		50% ± 30%
Where do you get the mosquito net? (Multiple answer question)	197	
Governmental institution		62 (31.5%)
Market		143 (72.6%)
Un-governmental organization		5 (2.5%)
Others		5 (2.5%)
Did anyone sleep under the mosquito net from the household member during the past 12 months? (Yes)	197	148 (75.1%)
Did everyone sleep under the mosquito net from the household member during the past 12 months? (Yes)	148	67 (45.3%)
If some or all household members didn't sleep under the mosquito net during past 12 months, please provide the reasons for not using it	127	
Financial problems		11 (8.7%)
We do not like it		17 (13.4%)
Laziness		13 (10.2%)
Allergy		11 (8.7%)
Breathing difficulties		23 (18.1%)
Unavailable bed nets		15 (11.8%)
Not needed		18 (14.2%)
Others		30 (23.6%)
If not all household members are not sleeping under the mosquito net, please mention the number of household members who are sleeping under bed net:	81	
0–3		51 (62.9%)
4–6		28 (34.6%)
7–9		2 (2.5%)
Percentage of household members who are sleeping under bed net (if not all household members were sleeping)	81	
Mean ± SD		40% ± 20%

^1^Data were presented as n (%); Mean ± SD

### Reasons for not having mosquito nets

Among households without bed nets in their houses (36.5%), the main reasons for not having them were the unavailability of bed nets in their area (38.9%), followed by not liking bed nets (30.1%), and therefore they did not have them in their houses (Table 5).

**Table 5:** Reasons for not having bed nets in households

Variables	N	N = 310^1^
What are the reasons for not having mosquito nets in your house?	113	
Unavailable		44 (38.9%)
High cost		27 (23.9%)
We do not like it		34 (30.1%)
Others		30(24.4%)

^1^Data were presented as n (%)

### Practices of preventives measures against malaria

Some participants (42.9%) mentioned that they used mosquito coil/repellents to prevent a malaria infection, while 41.9% used insecticide-treated bed nets. Only 35.8% of participants stated that their house was sprayed with insecticide chemicals in the past year. Among households sprayed with insecticide chemicals, 86.4% of them sprayed all rooms in the house (Table 6). Households with bed nets were more likely to spray their houses compared with those without (P = 0.005) (Table 6).

**Table 6:** Practice of preventive measures in households having and not having bed nets

Variables	N	Overall, N = 310^1^	Presence of bed nets within the household		p-value^2^
			Yes, N = 197^1^	No, N = 113^1^	
What are the preventive measures for malaria you use in your house?					
Untreated bed nets		77 (25.0%)	77 (39.3%)	0 (0.0%)	** < 0.001**
Insecticide-treated bed nets		116 (37.7%)	116 (59.2%)	0 (0.0%)	** < 0.001**
Window nets		84 (27.3%)	55 (28.1%)	29 (25.9%)	0.7
Using insecticide based repellents sprays		99 (32.1%)	67 (34.2%)	32 (28.6%)	0.3
Cleaning of positive mosquitoes breeding and resting places		98 (31.8%)	60 (30.6%)	38 (33.9%)	0.5
Using mosquito coil/repellents		132 (42.9%)	89 (45.4%)	43 (38.4%)	0.2
Not using		45 (14.6%)	10 (5.1%)	35 (31.2%)	** < 0.001**
Was your house sprayed with chemicals to protect your family from getting malaria in the last 12 months? (Yes)	310	111 (35.8%)	82 (41.6%)	29 (25.7%)	**0.005**
If yes, were all room sprayed? (Yes)	110	95 (86.4%)	72 (88.9%)	23 (79.3%)	0.2

^1^Data were presented as n (%)

^2^Fisher’s exact test; Pearson’s Chi-squared test

Bold numbers in the p-value column mean they are statistically significant

## Discussion

The overall treatment-seeking behaviour, awareness, and preventative practices toward malaria among participants living in rural areas in Gezira state, Sudan was found good. Concerning awareness and knowledge, most participants had a good knowledge of the disease, its transmission routes and preventive methods. For treatment-seeking behaviour, the majority went to primary health centres to seek treatment (77.9%). However, there was a high number of malaria cases among the population, therefore continuous effective control, interventional programmes and campaigns should be offered to this population.

This community households-based study had a total of 1955 household members. In Gezira state, severe malaria cases are the major cause of hospital admission and constitutes a fifth of severe malaria cases in the entire country, accounting for almost 17% of total malaria deaths nationally [4]. Malaria is one of the deadliest parasitic infections. Previous studies have stated that insecticide-treated bed net (ITN) utilization reduced malaria-related mortality by 20% [13]. Another study in the Democratic Republic of Congo found that an ITN coverage programme in rural areas reduced the under-five mortality rate by 41% [14]. Bed nets are considered one of the most successful interventions against malaria. Even before treating insecticides with bed nets, they were used as a physical barrier to reduce malaria infection [15–17]. More than half the participants in this study (63.5%) mentioned that they have bed nets available for use in their household, but less than half (45.3%) stated that everyone in their household slept under the bed net in the past year. The main reason for not sleeping under bed nets was difficulty breathing while using them (18.1%), indicating that even people with bed nets do not use them. Bed nets should be used by people to prevent malaria, and people must be educated about the importance of using and not using bed nets. A positive correlation was found between malaria infection and incorrect usage of bed nets [18].

Regarding knowledge, participants showed a reasonable level of knowledge regarding malaria. All participants stated that they have heard about malaria, and this could be explained by the high endemicity of malaria and the presence of one case of malaria in the past year in most households. This finding was consistent with studies from Mozambique, Tanzania and Swaziland [19–21]. The majority of participants (75.8%) identified the symptoms of malaria, which is important for recognition of cases and early treatment-seeking behaviour for affected individuals. Similarly, in research conducted in eSwatini, 70% were able to identify symptoms [21]. The minor difference might be due to the less exposure to malaria in eSwatini, and hence less experience regarding symptoms of the disease.

Participants were knowledgeable about preventive measures for malaria, including usage of bed nets (60%), indoor residual spraying (42.2%), and drainage of stagnant water. Knowledge of these protective measures is important for individuals to protect themselves and their families from the threat of malaria infection in such endemic areas, and to decrease the burden of malaria in their community by taking steps towards elimination of the disease.

Concerning treatment-seeking behaviour, the majority reported that they went to primary health centres for treatment (77.9%). Most went on the first day of being aware of symptoms (69.1%). This is assumed to be a reasonable percentage due to socio-economic and education status of people in the area, which were reflected in patients by increasing their awareness about the importance of seeking treatment to avoid severe debilitating complications. In a study conducted in Nigeria [22], socio-economic status (SES) disparities in health service utilization for malaria treatment were found in all communities studied. The results showed that the least poor SES group was more likely to seek treatment when ill than the poorest SES group. Thus, the least poor SES group is less likely to hesitate before seeking treatment than the poorest SES group. The results also show that the distance of the healthcare provider was a strong determinant of where people first sought malaria treatment. Easy availability of medications was the second most important factor. Quality of service was also an important factor. Nigeria, like Sudan, is a malaria-endemic area. Difficult economic status in Nigeria and many sub-Saharan African countries prohibits household members, especially those from poor backgrounds, from seeking or delaying formal healthcare, contributing to the high mortality and morbidity rates of malaria. And just as in Sudan, Nigerians have a range of treatment options when they are ill. These include public health facilities and formal and informal private sector health facilities [23]. The seemingly never-ending economic difficulties has led to a surge in informal private sector treatment options [24]. These findings were consistent with a study in eSwatini, where most respondents seek treatment in the health facilities [21]. The majority (88.1%) of respondents in this study stated that they would seek treatment within 24 h of onset of malaria symptoms. This higher percentage could be due to the better accessibility to health facilities and high socio-economic and educational status of the community. Another possible major reason for delaying treatment could be the use of traditional medicine, as found in low to middle-income Asia–Pacific countries [25]. A study conducted in a rural area of Madagascar found that only 28.7% sought care, and most of them visited public health facilities 46.8%, followed by community healthcare volunteers 31.0% [26].

Interestingly, most of this study’s participants (94.8%) stated that they had a history of malaria in their households in the past 12 months. The findings revealed that treatment-seeking behaviour, awareness and preventive practice toward malaria were reasonable and inconsistent with the high prevalence of malaria. Regarding this high prevalence, the assumption is that there are additional factors contributing to the high prevalence of malaria, such as the agricultural nature of the area and the lack of bed nets in the area's market, as was found in the results.

This study contains several strengths: the community-based study was the first study on this topic in Abu Ushar, Gezira state, Sudan, with different parameters being assessed among households. The sample size was representative as it covered 310 randomly selected households; this made the results more accurate. The findings may help the Ministry of Health in scaling up efforts to enhance malaria knowledge and complement with other preventive strategies that are being implemented at the national level.

There were limitations which can be summarized:. firstly, the study was done on at household level by interviewing only the head of the household, not every member of the house. Secondly, the sample may not represent all cities in Sudan, only Abu Usher, Gezira state. Lastly, this study was cross-sectional in design. It measured the previous malaria infection, not the current active malaria infection, by asking the head of household. The nature of this study design prohibits assessing the change in malaria infection over time after using various protective methods such as mosquito nets. Hence, a follow-up study is required to determine the change in malaria prevalence due to bed nets and other protective methods.

## Conclusion

The findings of this study indicated that households in Sudan had good knowledge of malaria, its transmission routes and prevention measures. However, there is a high prevalence of malaria among households. Therefore, continuous effective control of malaria, interventional programmes and campaigns should be offered to this population. Increasing the awareness in the community concerning malaria through educational programmes by teaching them about different malaria symptoms, stressing the importance of seeking treatment immediately, and teaching about various preventive methods, especially the proper usage of bed nets is recommended. Providing this area with bed nets and anti-malarial drugs due to their positive impact on malaria and reducing the prevalence of malaria is advised. There is a need to improve the availability of information about malaria through rural dispensaries and primary health centres, with special attention given to illiterate community members.

## Data Availability

The datasets used and/or analyzed during the current study are available from the corresponding author on reasonable request.
